# Sleep medicine in Saudi Arabia: Current problems and future challenges

**DOI:** 10.4103/1817-1737.74269

**Published:** 2011

**Authors:** Ahmed S. BaHammam

**Affiliations:** *University Sleep Disorders Center, Department of Medicine, College of Medicine, King Saud University, Riyadh, Saudi Arabia*

**Keywords:** Developing countries, Saudi Arabia, sleep, sleep centers, sleep disordered breathing, sleep laboratories, sleep medicine

## Abstract

Sleep medicine is a relatively new specialty in the medical community. The practice of sleep medicine in Saudi Arabia (KSA) began in the mid to late nineties. Since its inception, the specialty has grown, and the number of specialists has increased. Nevertheless, sleep medicine is still underdeveloped in the KSA, particularly in the areas of clinical service, education, training and research. Based on available data, it appears that sleep disorders are prevalent among Saudis, and the demand for sleep medicine service is expected to rise significantly in the near future. A number of obstacles have been defined that hinder the progress of the specialty, including a lack of trained technicians, specialists and funding. Awareness about sleep disorders and their serious consequences is low among health care workers, health care authorities, insurance companies and the general public. A major challenge for the future is penetrating the educational system at all levels to demonstrate the high prevalence and serious consequences of sleep disorders. To attain adequate numbers of staff and facilities, the education and training of health care professionals at the level of sleep medicine specialists and sleep technologists is another important challenge that faces the specialty. This review discusses the current position of sleep medicine as a specialty in the KSA and the expected challenges of the future. In addition, it will guide clinicians interested in setting up new sleep medicine services in the KSA or other developing countries through the potential obstacles that may face them in this endeavor.

Although sleep medicine is considered a relatively new specialty, interest in sleep and sleep disorders has existed since the beginning of mankind. Sleep is mentioned frequently in the Holy Quran, including a verse that says, “And among his signs is your sleep by night and by day” (Sūrah 30, Ar-rūm, verse 23). Early descriptions of two of the main sleep pathologies appeared in the 19^th^ century. The first was the description of narcolepsy by Jeane Baptiste Edouard Gélineau in 1880, and the second was the description of the main sleep disorder, obstructive sleep apnea (OSA), in 1836, not by a clinician but by the novelist Charles Dickens.[1] Sleep apnea was described later by clinicians in 1965, marking the most important advancement in the history of sleep medicine.[2,3] Basic research on the physiology of sleep medicine expanded between the 1930s and 1970s, when researchers explored the changes and mechanisms of sleep in animals, which facilitated the understanding of the major elements of sleeping brain waves.[4–8] The above discoveries were the foundation for the evolution of sleep medicine into clinical practice as a specialty. The world’s first sleep disorders clinic was launched at Stanford University in 1970.[1] The introduction of the continuous positive airway pressure (CPAP) technique as an effective treatment for OSA in 1981 resulted in a rapid increase in the interest in sleep medicine and the number of specialized centers and physicians practicing sleep medicine.[9] At present, the professional contents of sleep medicine are sufficient to justify the recognition of sleep medicine as an independent specialty. In 2005, sleep medicine was approved as an independent specialty in the USA and Germany.[10] In the Kingdom of Saudi Arabia (KSA), the first certification exam for sleep medicine as an independent specialty was approved by King Saud University in 2009.

In the early and mid-1990s, pulmonologists in the KSA used overnight pulse oximetry to diagnose OSA and titrated CPAP blindly to comfort patients and eliminate repetitive dips in oxygen saturation during sleep.[11–13] The use of proper “type I” full polysomnography in the KSA started relatively recently. Two hospitals in Riyadh started performing overnight sleep studies in the late 1990s (Riyadh Kharj Hospital and King Khalid University Hospital). At that time, the main focus of both hospitals was OSA. In the new millennium, sleep medicine service in the KSA has improved through the opening of a number of sleep disorders facilities in different regions of the country; nevertheless, the service is still in its early stages and faces many challenges.

This review aims to discuss the current position of sleep medicine as a specialty in the KSA and the expected challenges of the future by utilizing locally published data and data from other countries. In addition, it will help clinicians interested in setting up new sleep medicine services in the KSA or other developing countries to recognize the potential obstacles that may face them in this endeavor.

## Size of the Problem

Studies that have addressed the prevalence of sleep disorders in the KSA are limited. However, based on available data and waiting lists for sleep studies,[14] it appears that sleep disorders are prevalent among Saudis. Using the Berlin questionnaire to assess the prevalence of OSA risk and symptoms among middle-aged Saudi men and women in their primary care setting revealed that 3 out of 10 Saudi men and 4 out of 10 Saudi women are at a high risk of OSA.[15,16] Another study conducted among Saudi patients admitted to the coronary care unit with acute coronary syndrome assessed sleep-disordered breathing objectively using type II comprehensive unattended polysomnography during the acute event and six months later. This study revealed that 56% of the studied patients had OSA (apnea hypopnea index ≥10/h).[17] Obesity is a major risk factor for OSA in general and in women in particular.[18] A number of studies have shown that obesity is prevalent among Saudis of both genders and among different age groups.[19–21] A nationwide survey conducted between 1995 and 2000 reported a 50.2% prevalence of obesity (body mass index (BMI) ≥30 kg/m ^2^) in Saudi women between 40 and 49 years.[19] The prevalence of OSA among Saudi women seems to be higher than that reported in other countries.[15] A study that assessed gender differences in OSA among Saudis reported that Saudi women who were referred to a sleep disorders center with clinical suspicion of OSA were older, more obese and presented with insomnia more frequently than Saudi men.[22] In addition, studies have suggested a more than 10-year delay between symptom onset and referral to sleep disorders centers in Saudi women with OSA,[22,23] which supports the belief that OSA is under-recognized and under-diagnosed in women, resulting in a significant delay in diagnosis and treatment. This delay may result in accumulated damage to the cardiovascular system.[24]

The prevalence of other sleep disorders has not been well explored among Saudis. Snoring has been reported in 17.9% of elementary school children.[25] The estimated prevalence of narcolepsy is 40/100,000 Saudis.[26,27] A recent study reported the prevalence of restless legs syndrome to be 5.2%.[28]

## Current Position of Sleep Medicine Service

A recent national survey quantitatively assessed sleep medicine service in the KSA.[14] It revealed that sleep medicine is underdeveloped in the KSA compared to developed countries. The survey identified nine sleep disorders facilities; seven were defined as sleep disorders centers that provide clinical diagnostic and therapeutic services for patients with different sleep disorders, and two were defined as sleep laboratories that provide diagnostic and therapeutic services limited to sleep-related breathing disorders such as OSA.[14,29] Only two hospitals reported having pediatric sleep medicine specialists, and four facilities reported having the needed setup to perform sleep studies for children less than four years old.[14]

Administratively, all surveyed sleep disorders facilities are under pulmonary medicine services.[14] Sleep medicine has become almost a subspecialty of pulmonary medicine in the past few years (50.8 and 43.8% of diagnostic sleep laboratory directors are pulmonologists in the US and Japan, respectively).[24,30–32] The discovery of an effective treatment for OSA (CPAP therapy) and the increased recognition of OSA and its serious complications have attracted pulmonologists to this new field. Nevertheless, sleep medicine remains an interdisciplinary field crossing different specialties and should be accessible to practitioners from other related specialties, particularly neurology and psychiatry.[33]

The per capita polysomnography rate in the KSA was 7.1 per year per 100,000 people, compared to 18.3–427 in developed countries [Table 1].[14,30,31,34,35] The number of beds designated for sleep studies per 100,000 people was 0.06 in the KSA compared to 0.3–1.5 in developed countries.[14] Despite the limited number of beds for sleep studies, the overall occupancy rate was 45.7%. While the occupancy rate was 61.1% in government hospitals, the occupancy rate in private hospitals was very low (18.0%).[14] Possible explanations for the low occupancy rate include the lack of an adequate number of trained sleep technologists who can run a full service everyday for the whole year. The very low occupancy rate in the private sector could be attributed to the fact that most insurance companies do not cover the cost of polysomnography in the KSA.

**Table 1:** Quantitative assessment of sleep medicine activity in Saudi Arabia compared to selected countries

Country	Population	No. of sleep facilities	No. of sleep beds	No. of beds/100,000	No. of studies/yr	No. of studies/ yr/100,000
Saudi Arabia[14]	21,500,000	9	14	0.06	1536	7.1
United States[31]	280,000,000	1,292	–	–	1,170,000	427.0
Canada[35]	31,400,000	100	440	1.4	116,000	370.4
Australia[35]	18,970,000	65	244	1.3	53,500	282.0
Belgium[35]	10,000,000	50	150	1.5	17,716	177.2
Spain[34]	40,341,462	63	--	0.29	17,270	45.6
United Kingdom[35]	58,800,000	84	170	0.3	25,000	42.5
Japan[30]	126,686,000	146	–	–	23,184	18.3

## Current Obstacles Facing the Practice of Sleep Medicine

**Figure 1:**
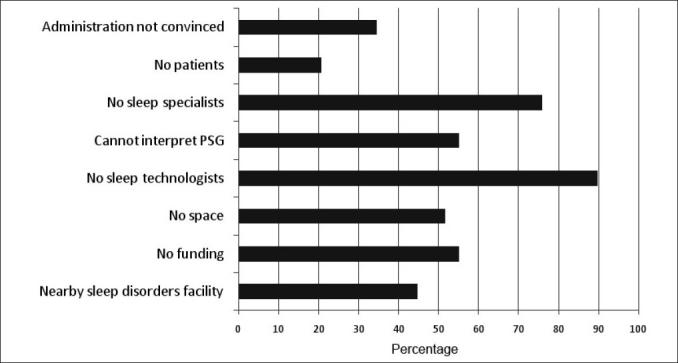
The most important reasons for not having a sleep disorders facility in hospitals that do not have sleep medicine service in Saudi Arabia. More than one reason was possible.[14] PSG: polysomnography

The practice of sleep medicine has grown significantly worldwide over the past two decades. The number of sleep centers and laboratories accredited by the American Academy of Sleep Medicine (AASM) has increased four-fold in the past decade.[31,36] This growth and the recognition of sleep medicine in developed countries can be attributed to a number of factors, including the recognition of an increasing number of sleep disorders, the increased evidence linking sleep disorders to serious medical problems, the availability of training programs for sleep medicine and the increased awareness of the general public about sleep disorders and their consequences.[37] Although sleep medicine has moved forward over the past decade in the KSA, major obstacles still face the specialty and practitioners [Figure 1]. Those obstacles can be categorized as follows:

### Inadequate number of qualified specialists

The number of sleep medicine specialists in the KSA is relatively low. The number of trained qualified sleep medicine specialists in the KSA is reportedly 19 physicians located in a few hospitals in three major cities.[14] This number is extremely low for a country the size of KSA. In addition, all sleep medicine specialists deal with sleep disorders as a small part of a larger medical practice, such as pulmonology or neurology. Establishing a good sleep medicine service requires a dedicated sleep medicine physician who has protected time for practicing sleep medicine. Another challenge that faces sleep medicine in the KSA is the lack of a reference body that can license sleep medicine specialists to practice sleep medicine based on their professional competence in the field. As a result, non-specialists with limited knowledge and experience in sleep medicine have started practicing sleep medicine, particularly in the private sector, which may reflect poorly on patient care.

### Shortage of trained sleep technologists

A shortage of trained sleep technologists is a global problem, and in the KSA, this is considered the major obstacle facing sleep medicine.[14] A national survey revealed that the greatest bottleneck is lack of trained sleep technologists.[14] More than 80% of the surveyed hospitals stated that a lack of trained sleep technologists who can perform polysomnography was a major obstacle that prevented the establishment of a sleep medicine service.[14] To overcome this obstacle, formal training programs for sleep technologists and the establishment of a national registration exam are necessary. Meanwhile, intensive workshops should be organized to improve the skills of existing technologists and those who are planning to join the sleep medicine technology field. Good incentives must be given to technologists from respiratory therapy backgrounds to join this specialty.

### Knowledge and attitude of health care workers and the general public toward sleep medicine

Sleep disorders and sleep medicine as a specialty are under-recognized by both the public and health care workers. The KSA public accepts information on sleep disorders from any source without criticism, even if the information is not authentic; therefore, they have developed their own ideas and myths about sleep. Some of the public think that sleep problems are part of their nature and do not think of these problems as medical issues that can be treated. They do not perceive sleep disorders to be as critical as other health problems and do not know about the serious comorbid conditions associated with sleep disorders. In addition, many patients do not know to which specialty they should go to present their sleep problems. Most patients seen in the clinic have gone to many doctors, particularly psychiatrists, before attending the sleep disorders clinic, thinking that all sleep disorders are handled by psychiatrists. In addition, practitioners face problems persuading patients to modify their sleep pattern or to apply good sleep hygiene. Due to under-recognition of the seriousness of disorders like OSA, patient compliance with CPAP in the KSA is less than that reported in developed countries, even when they have good objective and subjective responses to CPAP in the sleep disorders center.[38]

On the other hand, the knowledge of practicing physicians, particularly primary care physicians, about sleep disorders is limited.[39] In general, medical students in the KSA rarely have the chance to learn sleep medicine in medical schools. Similarly, postgraduate teaching of sleep disorders during residency training seems to be limited as well. This lack of education and training in sleep medicine has resulted in a culture of physicians who have very limited knowledge about sleep disorders and, as a result, are likely to under-diagnose and under-treat sleep disorders. A survey of primary health care (PHC) physicians in all primary care centers in Riyadh revealed that PHC physicians do not completely recognize the importance and impact of OSA and other sleep disorders.[39] Forty-three percent of the participants did not realize the existence of sleep medicine as a specialty, 40% felt that sleep disorders are not common and 38% did not know to whom they should refer their patients.[39] Their recognition of some of the serious consequences of OSA was poor.[39] In addition, the recognition of other sleep disorders seems to be low in the KSA. One study reported that 53.2% of narcolepsy patient referrals to the sleep disorders clinic were patient initiated.[26] Among those referred by physicians (46.8%), only 6.4% of patients were referred with the correct diagnosis.[26] Moreover, the interval between symptoms onset and diagnosis was more than 8 years.[26] The delayed diagnosis cannot be attributed to the lack or reduced access to the health care services as the diagnosis was missed by the treating clinicians in most of the studied patients.[26] Another study reported that 19.4% of patients with insomnia were referred by their primary physician to the sleep disorders clinic; the remaining were patient-initiated referrals.[40] A third study reported that referrals by otolaryngologists represented 8% of OSA patients, compared to 17.4% in the US.[41,42] The health system in the KSA relies on the referral system, where the patient’s first exposure is usually to the PHC physician, who assesses and decides the patient’s plan of management. Thus, early detection and management of patients with sleep disorders depends considerably on the knowledge and awareness of PHC physicians. As PHC physicians have limited knowledge about sleep disorders, it is likely that sleep disorders among PHC attendees will be under-recognized and that patients with these disorders may be labeled with inaccurate diagnoses and may receive inappropriate treatment.[43] Studies in the KSA and Western countries have shown that OSA is common among patients attending PHC clinics.[15,16,44] With the limited number of sleep medicine specialists in the KSA, it is impractical to expect that they will be able to be the primary caregivers for all patients with sleep disorders. Therefore, an alliance of sleep medicine specialists, PHC physicians and general physicians (internal medicine and pediatrics) becomes essential. Educational interventions are effective in increasing the rates of recognition of OSA among PHC physicians.[45] In addition, a study has demonstrated that patient compliance rates with CPAP therapy managed by their PHC physicians was comparable to patients managed by sleep medicine specialists.[46]

### Health care authorities and insurance companies

Unfortunately, sleep medicine is not considered among the priorities or core competencies of decision makers in some hospitals. A national survey conducted recently revealed that “unconvinced administration” was one of the main obstacles facing the establishment of sleep disorders facilities in some hospitals.[14] In addition, the private sector did not invest much in this specialty, as most insurance companies do not cover the cost of performing sleep studies or treating OSA. Hence, sleep medicine specialists have to communicate a clear and evidence-based message to decision makers and insurance companies indicating the high prevalence of sleep disorders in the community, the associated serious comorbid conditions and their impact on morbidity and mortality.[47] Decision makers should understand the impact of early diagnosis and treatment of sleep disorders on morbidity, mortality and cost savings. There is good evidence supporting the reduction in morbidity and mortality of OSA patients when CPAP treatment is initiated.[48] Moreover, treatment of sleep disorders has been shown to decrease health care utilization and costs paid by health care payers.[49–61] Due to the established effects of CPAP, many countries now recommend that CPAP therapy should be available to patients with symptomatic OSA.[62,63]

The lack of designated sleep disorders facilities may result in admitting patients with sleep disorders into valuable hospital beds that could be used to treat acutely ill patients who need inpatient care. Bahammam and Rahman demonstrated that in the absence of proper sleep disorders facilities, patients with OSA spend 4.5 nights on average as inpatients for the sake of CPAP titration, which costs around SR6750 per patient.[11] A few studies have shown that the outcome of patients with sleep disorders is better when managed by sleep medicine specialists and in proper, accredited sleep disorders facilities.[64,65] Therefore, we need to demonstrate to decision makers and policy makers that patient outcome is better when managed by qualified specialists in proper sleep medicine facilities, and we need to emphasize the cost effectiveness of the provided service.[66,67]

### Diagnostic equipment and after-sale service

One major obstacle that faces practitioners in developing countries is the after-sale service.[68] The performance of polysomnography and the process of data acquisition and scoring are complicated and require expertise. Some local agents of sleep-diagnostic systems promote their machines as “plug-and-play” devices with reliable software that can clean the signal and score the data. This has resulted in having non-functioning sleep-diagnostic systems in a number of hospitals. Often, local suppliers do not provide efficient after-sale service and do not participate actively in hands-on training because of either staff shortages or a lack of adequate training and knowledge of the product sold. Therefore, before establishing a sleep disorders facility, the involved team should clearly define their needs with regard to the type of sleep studies to be performed, the number of needed channels and the number of beds in the new facility. Defining the specifications of different brands and choosing the system that will best suit their needs is important. However, having a local reference for the diagnostic system is more important. International references may not reflect the local experience in developing countries.

## Future Direction

### Education

Education should be addressed at two levels: level I, education of medical and technical staff; and level II, education of the general public.

#### Education of physicians and technologists

There is no doubt that sleep disorders are under-recognized by practicing physicians. In a study in the US that examined more than 1,000,000 patient records, only 17 positive diagnoses of sleep disorders were made, which is less than 1/1000 of the expected number of sleep disorders based on the current estimates.[69–71] In general, most physicians receive no or minimal education about sleep medicine during medical school or residency training,[39,72] which may compromise patient care. The Saudi boards for pulmonary medicine (adult and pediatric) and psychiatry have recently begun to address this issue and send their trainees for formal training in sleep medicine. However, other specialties, such as internal medicine, neurology, otolaryngology and primary care, need to do more to address this issue. We need to reach out to the other specialties to demonstrate the importance of both theoretical and practical training in sleep medicine for trainees to be able to diagnose, treat and refer patients to sleep specialists if needed. In addition, it is hoped that medical schools will provide adequate education in sleep medicine.

The education and training of physicians to become specialists in sleep medicine is another challenge. The number of qualified sleep medicine specialists in the country is very low and does not meet the increasing demand for service.[14] The need for local fellowship training programs in sleep medicine is highly needed in face of the increasing demand and difficulty of finding acceptance for formal training in developed countries. King Saud University has made a major step forward by launching the King Saud University Fellowship in Sleep Medicine. However, more training programs are needed to meet the expected demand. Therefore, the Saudi Commission for Health Specialties has to adopt and support this new specialty and launch an interdisciplinary Saudi Board in Sleep Medicine for adults and children.

Since the early 1970s, polysomnographic technologists have been the technical group trained to perform polysomnography for the diagnosis and treatment of sleep/arousal disorders, including the management of CPAP titration for OSA. In the KSA, sleep technologists usually come from a respiratory therapy, nursing, or electroencephalography technology background.[14] To work independently, sleep technologists need intensive hands-on training and experience and a thorough knowledge of the technical aspects of data acquisition, analysis and sleep-induced changes in the physiology of various body systems, including, but not limited to, the neurological, musculoskeletal, cardiac and respiratory systems. Currently, there are no programs in the KSA that graduate qualified sleep technologists. Therefore, those interested in sleep technology and sleep technologists who want to improve their knowledge and skills should attend short, intensive courses and workshops about polysomnography. Training in sleep centers that have good experience in sleep technology is another effective solution. Eventually, we need to have a registration exam for sleep technologists that ensures the highest level of competence and expertise in the field of polysomnography.[73]

#### General public awareness

Despite individual efforts by some sleep specialists to educate the public about sleep disorders through different media channels, the majority of the general public remains unaware of the serious consequences of sleep disorders, sleep deprivation and disturbances of biological rhythms. Sleep deprivation is a major problem among youths in the KSA.[74,75] Many patients with sleep disorders tend to quickly lose motivation during the treatment of different sleep disorders, and many patients with sleep-disordered breathing do not accept the use of PAP devices.[38] Therefore, we need a collaborative effort between sleep medicine specialists, probably through the Saudi Sleep Medicine Group (SSMG) and the Saudi Thoracic Society (STS), to reach patients through different channels of the media and to organize educational programs targeting the patients and forums for patients with different sleep disorders.

## Accreditation

Specialty care has been shown to improve outcomes in several settings. For example, a number of studies in intensive care units have demonstrated that management of critically ill patients by qualified critical care specialists resulted in a reduction of mortality, hospital stay and health care utilization.[76–78] Similar results have been demonstrated in patients with heart failure who were managed by cardiologists rather than internists.[79] In a recent study, Parthasarathy and colleagues demonstrated that accreditation and certification status of sleep centers and physicians by the AASM was associated with better outcomes in patients with OSA.[64] Voluntary accreditation of sleep medicine facilities has begun in some countries like the USA in 1976 and Germany in 1989.[80] The European Sleep Research Society recently published guidelines for the accreditation of sleep medicine centers, and the AASM published standards for the accreditation of sleep disorders centers and laboratories for sleep-related breathing disorders.[80–82]

The accreditation process aims to ensure that the facility and its staff meet the highest quality standards. The accreditation standards describe the required structural, professional and human resources, clinical and technical standards, and emergency and quality assurance methods.[81,82] The practice of sleep medicine in the KSA needs to be organized. Accreditation of sleep disorders facilities is needed. The SSMG and the STS could be good groups to initiate such a system.

The accreditation process should assess the credentials of medical and technical staff. Therefore, there should be a licensing system to license sleep medicine specialists and sleep technologists. The Saudi Commission for Health Specialties should take on this role and develop guidelines and requirements for the licensing of medical specialists and sleep technologists. Nevertheless, the SSMG can initiate the process through direct communication with the Saudi Commission for Health Specialties to develop defined standards for the required education and training and the need for formal evaluation.

## Research

Although the last three decades have witnessed significant growth and evolution in sleep research worldwide, sleep research remains underdeveloped in the KSA, which mirrors the underdevelopment in the clinical service overall. In 1994, the KSA published 10 papers on sleep and was ranked number 31 in the world in terms of number of publications.[83] Although the number of publications increased in 2004 to 17 papers, our ranking decreased to 39.[83] Regionally, the KSA was ranked fourth after Turkey, Israel and Iran.[83] It is obvious that more sleep research is needed, particularly research that addresses the prevalence of different sleep disorders in the KSA. Such research is needed to demonstrate to health care providers and decision makers the size of the problem and to help strategic health planners estimate the number of sleep specialists and sleep facilities needed to meet the increasing demand. Academic centers should develop research programs for clinical and basic sleep research. Collaboration among local centers and affiliations with internationally renowned research centers will reflect positively on the volume and quality of published work.

## Conclusions

Sleep medicine in the KSA is underdeveloped at the levels of practice, education and research. It faces a number of obstacles that hinder its progress, including a lack of adequate specialized medical and technical staff and a lack of awareness about sleep disorders and their serious consequences among health care workers, health care authorities, insurance companies and the general public. A major challenge for the future is penetrating the educational system at all levels to demonstrate the high prevalence and serious consequences of sleep disorders. Educating and training health care professionals at the level of sleep medicine specialists and sleep technologists is another important challenge that faces the specialty to attain an adequate number of staff and facilities. Sleep research is mandatory to assess the size of the problem in the KSA and to help strategic health planners estimate the number of sleep specialists and sleep facilities needed to meet the increasing demand.
